# Prediction of Duration of Traffic Incidents by Hybrid Deep Learning Based on Multi-Source Incomplete Data

**DOI:** 10.3390/ijerph191710903

**Published:** 2022-09-01

**Authors:** Qiang Shang, Tian Xie, Yang Yu

**Affiliations:** School of Transportation and Vehicle Engineering, Shandong University of Technology, Zibo 255000, China

**Keywords:** duration prediction, deep learning, traffic data fusion, hybrid network, intelligent transportation systems

## Abstract

Traffic accidents causing nonrecurrent congestion and road traffic injuries seriously affect public safety. It is helpful for traffic operation and management to predict the duration of traffic incidents. Most of the previous studies have been in a certain area with a single data source. This paper proposes a hybrid deep learning model based on multi-source incomplete data to predict the duration of countrywide traffic incidents in the U.S. The text data from the natural language description in the model were parsed by the latent Dirichlet allocation (LDA) topic model and input into the bidirectional long short-term memory (Bi-LSTM) and long short-term memory (LSTM) hybrid network together with sensor data for training. Compared with the four benchmark models and three state-of-the-art algorithms, the RMSE and MAE of the proposed method were the lowest. At the same time, the proposed model performed best for durations between 20 and 70 min. Finally, the data acquisition was defined as three phases, and a phased sequential prediction model was proposed under the condition of incomplete data. The results show that the model performance was better with the update of variables.

## 1. Introduction

Road traffic injury (RTI) is still the leading cause of death and disability worldwide, causing about 13.5 million deaths annually [1]. RTIs are also the primary killers of people aged 5 to 29. Reducing traffic accidents is a global public security challenge. Traffic accident prediction and analysis are extremely important for optimizing public transport, building safer routes, and reducing traffic operation costs. Traffic incidents refer to non-repetitive incidents such as traffic accidents, vehicle stalls, overflow loads, temporary construction, and maintenance activities. In addition, it is easy to cause secondary incidents after traffic accidents, resulting in greater and wider negative impact. In consideration of its importance, the prediction and analysis of traffic incident duration have been studied extensively in the past decades.

**Figure 1:**
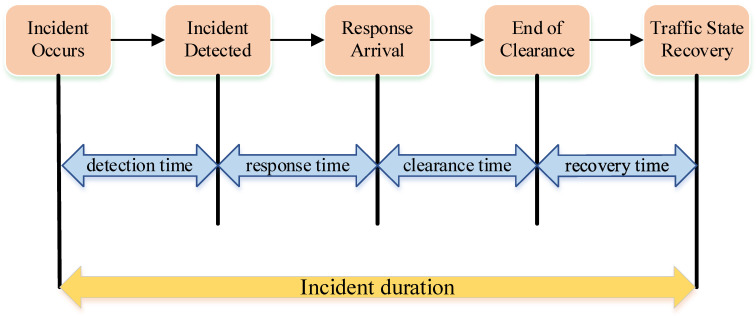
The composition of incident duration.

The traffic incident duration includes four parts: the detection time, response time, clearance time, and recovery time [2]. The automatic detection time of the traffic incident duration depends on the performance of the incident detection model. Response time refers to the time when the incident is detected and the incident handlers and rescuers arrive at the scene. Clearance time is the time consumed by incident handlers to clean up the scene and rescue personnel to carry out rescue operations. The recovery time is the time that the traffic state returns to normal. Figure 1 illustrates the composition of incident duration. If the time of these four parts can be shortened, the negative impact of the incident on traffic delays and road environment will be reduced. The randomness of traffic incidents and the uniqueness of each incident make the incident duration difficult to predict, but the traffic management center and traffic incident management system need accurate and reliable prediction results to guide the traffic information strategy and improve management efficiency.

### 1.1. Motivation

The purpose of this paper was to establish a traffic incident duration prediction model from the perspective of a traffic policymaker. The detection time and response time of the traffic incident duration are affected by the performance of the detection algorithm and the execution ability of the agencies, so we mainly studied the clearance time and recovery time. The duration mentioned later in this article refers to these two parts.

Over the past few decades, many methods have been proposed to predict the incident duration. In terms of the data sources and types, a large number of studies have focused on a small-scale dataset of several roadways or a region.

Won et al. presented a knowledge-based system based on detailed incident reports collected by the I-95 roadway between the years 2012 and 2016. The proposed system featured the use of interval-based estimates derived from knowledge of historical data, with different confidence levels for each estimated incident clearance duration [3]. Fu et al. proposed a multi-task learning framework to predict the impact of traffic incidents while predicting the duration and identifying the key time feature groups based on some traffic incidents that occurred on six main roads (I-270, I-295, I-395, I-495, I-66, and I-95) in the United States in 2018 [4].

Certainly, some scholars have used larger datasets, but most of these datasets are private or need other ways to obtain, which is difficult to obtain without permission.

In 2020, scholars proposed a model system that recognized the distinct traffic incident duration profiles based on incident types for 326,348 incidents collected in the Grand Orlando region from 2012 to 2017. Specifically, a copula-based joint framework was estimated with a scaled multinomial logit model system for the incident type and a grouped generalized ordered logit model system for the incident duration to accommodate the impact of observed and unobserved effects on the incident type and incident duration [5].

Zhang et al. studied whether a larger sample size would increase the reliability of the traffic incident duration prediction model in the case of Eastern Tennessee. A total of 47,331 records of 17 routes from 26 counties were collected from 1 January 2015 to 31 December 2016. This study estimated handfuls of hazard-based duration models with varying sample sizes. The relationships between the sample size and model performance, along with the estimate outcomes, were examined and visualized. The case study suggested a sample size of 6500 to be sufficient for a reliable incident duration model. However, this conclusion is based on the hazard-based duration model, and whether the data-driven machine learning algorithm is applicable remains to be studied [6].

At the same time, these studies did not provide information on the specific means of obtaining their data and how they were used, but in the end, thanked the relevant agencies for their data assistance.

As far as the datasets above-mentioned are concerned, they record some feature descriptions and environmental information about incidents such as incident ID, incident type, incident location, number of blocked lanes, duration, time in a day, one day in a week, and land use types. In recent years, with the rapid development of communication technology, 5G communication has gradually begun to be applied in private enterprises. Social media data such as Twitter and Facebook, and the natural language data of text types recorded by some road traffic real-time sensors have also been widely studied and applied to traffic incidents.

According to the recent research on several systems based on sensors and social network platforms to detect the traffic incident and monitor traffic conditions, a social network–based, real-time monitoring framework was proposed for traffic incident detection and condition analysis using ontology and latent Dirichlet allocation (OLDA) and bidirectional long short-term memory (Bi-LSTM) in 2021 [7]. The topic modeling method of OLDA was used to automatically label each sentence (e.g., traffic or non-traffic) from the social network to identify the exact traffic information. The FastText model and Bi-LSTM with SoftMax regression were trained for traffic incident detection and condition analysis. The proposed system is more efficient for traffic incident detection and condition analysis in comparison to other existing systems.

In 2018, Zheng et al. developed a data fusion framework to identify social media messages reporting non-recurring traffic incidents by connecting the traffic incidents with traffic states inferred from taxi global positioning system (GPS) data [8].

Considering the diversity of traffic incidents, it is more convenient to accept and store relevant data in the form of natural language than in constrained value fields. A prediction algorithm using Bidirectional Encoder Representations from Transformers (BERT) word embedding combined with LSTM regression was proposed in 2021. The prediction results of the traffic incident duration of LSTM, XGBoost, RF, and SVR were compared. The BERT-LSTM hybrid model could effectively capture the context meaning of the textual incident report and predict the traffic incident duration with an MAE around 11.16 min [9].

In addition, compared to the data fusion framework using natural language data, Li proposed a deep fusion model considering the temporal and spatial correlation of traffic flow, which can handle both categorical variables and continuous variables. In this model, a stacked restricted Boltzmann machine (RBM) was used to handle the categorical variables, a stacked Gaussian-Bernoulli RBM was used to handle the continuous variables, and a joint layer was used to fuse the extracted features. The results show that the fusion of different types of variables can improve the prediction accuracy [10].

In conclusion, due to the limitation of data scale in previous studies, this paper intends to use a unique, open, and large-scale traffic incident dataset—the countrywide traffic incident dataset of the United States including 2.25 million traffic incidents [11]. The dataset records the traffic incident information that includes numeric types and natural language descriptions. Moreover, the research of the scholars above-mentioned on the data fusion framework shows that feature fusion is of great significance for predicting the duration of traffic incidents. Therefore, we propose a hybrid deep learning model based on multi-source data. Benefiting from its unique gate control structure, LSTM has great advantages in prediction tasks and is widely used in various fields of prediction. In recent years, many articles about LSTM variants have been applied in traffic incidents. Therefore, the latent Dirichlet allocation (LDA) topic model in the proposed model was used to analyze and process the natural language data. Subsequently, the extracted data and numerical type data were input into the Bi-LSTM and LSTM hybrid models for training, and the trained model was regressed to predict the duration of traffic incidents using the test set.

Meanwhile, considering the difference in the time of traffic information acquisition, we divided the information acquisition into three phases. In the first phase, the information in the second and third phases is not available. When the incident state develops to the second phase, the information in the third phase is also unknown. This phenomenon is called the incomplete information condition. Based on this condition, we developed a phased sequential prediction model that could predict the duration of traffic incidents at each phase even without the information of the later phases, and this sequential prediction model has practical significance.

The next subsections provide a methodological overview of relevant research followed by our contribution.

### 1.2. Literature Review

Methodologically, traffic incident duration prediction methods can be divided into statistical methods and machine learning methods. Based on strict mathematical assumptions and functional structure, statistical methods are capable of explaining the mathematical relationship between estimators and explanatory variables. In contrast, machine learning models have more accurate modeling records for predicting the duration, but it is considered a black-box method because it provides little explanatory insight for the modeler. The details are as follows.

#### 1.2.1. Statistical Methods

Linear regression was the first method to solve prediction problems [12,13]. Then, a large number of regression models were gradually tried for application in the incident duration such as the log-linear regression model [14], ordinary least square regression, and truncated regression model [15].

Compared with traditional statistical algorithms, the hazard-based duration models are more accurate. Such models not only consider the effect of influencing factors on the length of the incident duration, but also care about the probability of the duration ending at the next short interval. Among them, the accelerated failure time (AFT) model has been widely used and many extended models have been developed [16,17,18].

In 2015, a competing risks mixture model was used to investigate the influence of clearance methods and various covariates on the duration of traffic incidents and predict the traffic incident duration [19]. Three candidate distributions, namely, generalized gamma, Weibull, and log-logistic were tested to determine the most appropriate probability density function of the parametric survival analysis model. Zou et al. introduced a quantile regression model in survival analysis to analyze the incident duration data in a more flexible way [20].

At present, the heterogeneity of incident duration is of wide concern. Wali et al. used a simulation-assisted random parameter and quantile regression models to explore the heterogeneous correlation between the incident duration and detection source, incident type, roadway type, temporal factors, and incident characteristics [21]. Islam et al. compared two advanced econometric modeling methods, random parameters duration modeling and latent class duration modeling, in understanding the factors that impact freeway incident clearance times. These two modeling approaches were further compared to identify which of them provided the best fit for the data with respect to accounting for unobserved heterogeneity. The results show that the latent class hazard-based model provided the better fit for the incident duration data [22]. In 2022, instead of using the variables extracted from incident descriptions and records, Chand et al. collected explanatory factors at a macro-level and used latent class models to estimate the crash duration and frequency for unobserved heterogeneity. The results showed that income, driver experience, and exposure are considered to have both positive and negative impacts on duration [23].

#### 1.2.2. Machine Learning Methods

Compared to the statistical methods, machine learning methods cannot demonstrate how various influence factors affect the prediction model. The same traffic information such as vehicle type, used in machine learning, may not contribute to other datasets. However, accurate duration prediction results have always been the key to the wide development of machine learning methods in the field of transportation. Because of their flexible structure, these methods can deal with the complex and highly nonlinear relationship between dependent variables and independent variables. Overall, machine learning methods can be divided into the neural network algorithm including deep learning [10], ensemble learning algorithm [24,25], support vector machine (SVM) [26], Bayesian network model [27], decision tree [28], etc.

In recent years, many scholars have reviewed previous machine learning models of event duration [29,30], in which Khaled retrieved more than 110,000 incident records with over 52 variables from the Houston TranStar data archive. Five machine learning algorithms including regression decision tree, SVM, ensemble tree, Gaussian process regression, and artificial neural network (ANN) were compared [31]. In 2022, Grigorev presented a novel bi-level machine learning framework enhanced with outlier removal and intra–extra joint optimization and found the optimal threshold between short-term versus long-term traffic incident duration. The final results indicate that the proposed approach significantly outperformed the baseline ML models in 66% of all cases [32].

However, most of the statistical and machine learning methods described above are based on the fact that all of the incident information is available, because these studies were conducted using historical datasets. In fact, due to various factors, it is almost impossible to obtain all the information, and incomplete information has become an important feature of traffic incident duration prediction. Therefore, it promotes the development of the dynamic incident duration model.

In 2020, Li compared the survival models of large-scale traffic incident duration and developed a sequential prediction method to deal with the temporal sequence process of incident information collection. Based on parametric survival modeling, a five-phase prediction method was proposed according to the time sequence of information available during emergency operation. The results showed that with the increase in information, the performance of the model will be improved according to the root mean square error and the average absolute percentage error [33]. In 2021, Zhu et al. used the multi-layer perception (MLP) and LSTM model to integrate the relevant factors of traffic incident and real-time traffic flow parameters to predict the duration. This framework was expected to help reduce the impact of incident on the road traffic efficiency and safety in future practical applications [34].

### 1.3. Our Contributions

In this subsection, our main contributions are summarized as follows.

1. A hybrid deep learning model for traffic incident duration prediction was proposed for the natural language data and sensor data. The LDA topic model was used to analyze and process the natural language data, and the Bi-LSTM and LSTM hybrid network was used for regression prediction;
2. Unlike previous study on the duration of a single road or a region, this study used a unique and large-scale countrywide traffic incident database to explore the characteristics of traffic incidents over a wider range of road networks, which is more universal;
3. This paper developed a phased sequential duration prediction model based on incomplete information, and explained how this phased prediction model was further developed into a dynamic prediction model.

The rest of this paper is organized as follows. Section 2 introduces the method used in this paper in detail. In Section 3, we describe the dataset used in this study as well as the model evaluation methods and comparison methods and their parameter settings. In Section 4, we analyze the experimental results and illustrate the construction method and results of the phased sequential prediction model. Finally, Section 5 presents a brief conclusion.

## 2. Methods

The proposed method is illustrated in this section.

### 2.1. LDA Topic Modeling

A latent Dirichlet allocation (LDA) model is a topic model that discovers underlying topics in a collection of documents and infers word probabilities in topics. The LDA was first developed by Blei et al. as a generative probabilistic modeling approach to reveal hidden semantic structures in a collection of textual documents [35]. The LDA is a three-level hierarchical Bayesian model, in which each item of a collection is modeled as a finite mixture over an underlying set of topics. Each topic is, in turn, modeled as an infinite mixture over an underlying set of topic probabilities.

A document is a sequence of N words denoted by $W=(w_{1},w_{2},w_{3},\ldots ,w_{N})$, where $w_{N}$ is the Nth word in the sequence. A corpus is a collection of M documents denoted by $D=(W_{1},W_{2},W_{3},\ldots ,W_{M})$. The LDA assumes the following generative process for each document W in a corpus D.

1. Choose N∼Poisson (ξ);
2. Choose θ∼Dir (α);
3. For each of the N words $w_{N}$:
  (a) Choose a topic $Z_{N}$∼Multinomial (θ).
  (b) Choose a word $w_{N}$ from $p(w_{N}\left|z_{N}\right.,\beta )$, a multinomial probability conditioned on the topic $Z_{N}$.

First, the dimensionality k of the Dirichlet distribution (and thus the dimensionality of the topic variable Z) is assumed to be known and fixed. Second, β represents the probability distribution of words in topics and θ represents the probability distribution of topics in documents. Finally, the Poisson assumption is not critical to anything that follows and more realistic document length distributions can be used as needed.

Obviously, $w_{N}$ is the only observable variable, and all of the others are latent in this model. Given the parameters α and β, the joint distribution of a topic mixture θ, a set of N topics Z, and a set of N words w are given by:

(1) $$p(\theta ,Z,w\left|\alpha ,\beta \right.)=p(\theta \left|\alpha \right.)\prod_{n=1}^{N}p(Z_{N}\left|\theta \right.)p(w_{N}\left|Z_{N},\right.\beta )$$

To determine the parameters α and β, Blei recorded a detailed calculation process in his paper. Here, we will not elaborate on this too much.

### 2.2. LSTM and Bi-LSTM

The LSTM is an advanced version of recurrent neural networks (RNN) proposed by Hochreiter and Schmidhuber in 1997. The LSTM network solves the long-term dependency problem that occurs in classical RNNs by introducing memory cells and the gate structure. Memory cells have self-connections that store the network temporal state and are controlled through four gates named as the input gate i, forget gate f, control gate c, and output gate o.

The core part of the LSTM is similar to that of a conveyor belt, which is generally called the cell state ($C_{t}$). It exists throughout the whole chain system of the LSTM. The forget gate decides which information from the input should be neglected from the previous memory and the input gate decides which information can be transferred to the cell, which can be defined as:

(2) $$f=sigmoid(W_{f}\cdot [h_{t-1},x_{t}]+b_{f})$$

(3) $$i=sigmoid(W_{i}\cdot [h_{t-1},x_{t}]+b_{i})$$

The control gate controls the update of the cell state from $C_{t-1}$ to $C_{t}$, based on Equations (4) and (5).

(4) $$C_{t}=f⊗C_{t-1}+i⊗\tilde{C_{t}}$$

(5) $$\tilde{C_{t}}=\tanh(W_{c}\cdot [h_{t-1},x_{t}]+b_{c})$$

The output gate calculates the predicted value and generates the new input of the next time slice, which needs to calculate the output of the hidden node $h_{t}$. This process can be defined as:

(6) $$h_{t}=o⊗\tanh(C_{t})$$

(7) $$o=sigmoid(W_{o}\cdot [h_{t-1},x_{t}]+b_{o})$$

In Equations (2)–(7), W and b represent the weights and bias variables, respectively. $h_{t-1}$ symbolizes the prior hidden layers node. Here, t−1 and t are the previous and current time steps, respectively.

**Figure 2:**
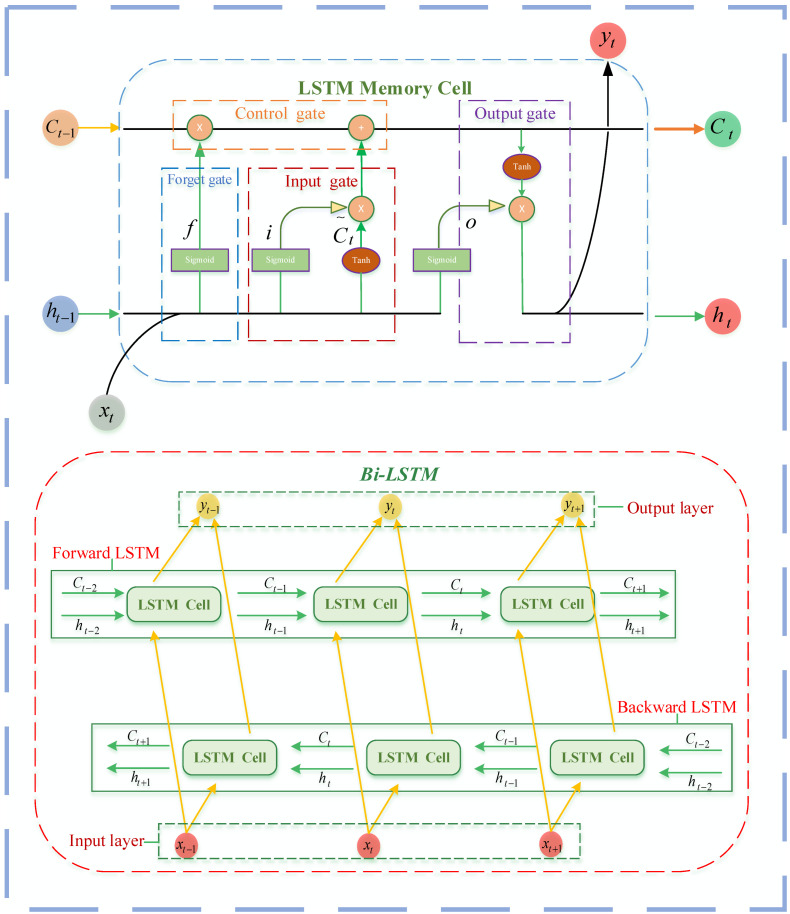
The general structure of the LSTM and Bi-LSTM.

The RNN and LSTM can only predict the output of the next moment according to the timing information of the previous moment. However, in some problems, the output of the current moment is not only related to the previous state, but may also be related to the future state. In order to overcome this limitation, the bidirectional recurrent neural (BRNN) network has been proposed. The output of the BRNN is determined by two similar results output from two recursive hidden layers in the opposite direction. Based on generative deep learning, the output layer can receive information forward and backward simultaneously. Thus, the Bi-LSTM is a network superimposed by two LSTM networks in the opposite direction of information transmission. Figure 2 shows the general structure of the LSTM and Bi-LSTM.

### 2.3. The Proposed Method

**Figure 3:**
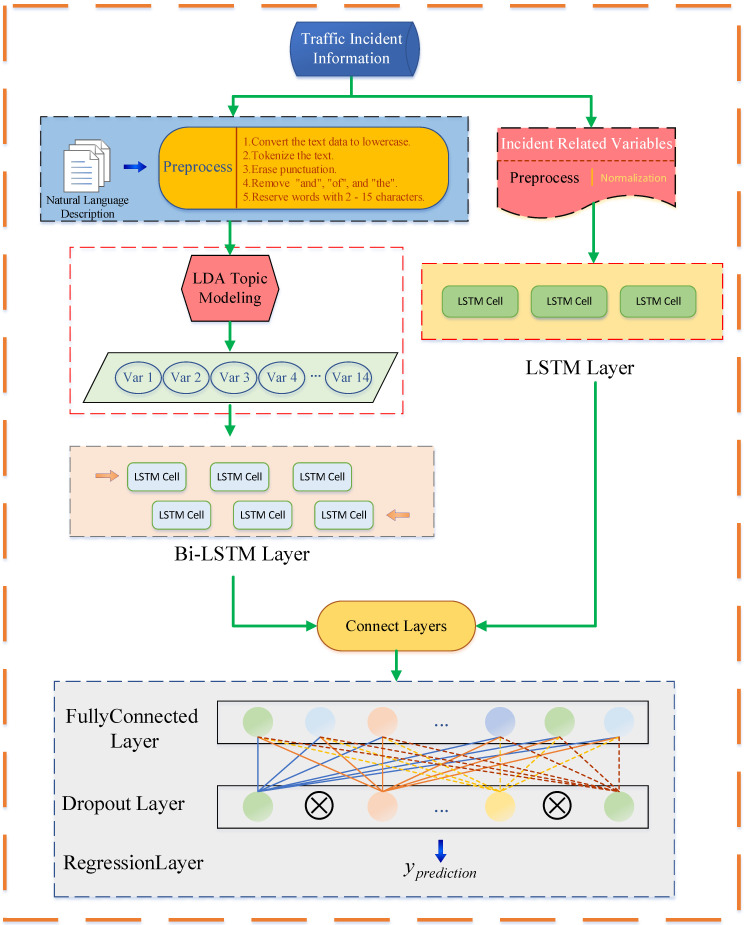
The structure of the proposed prediction model.

As mentioned earlier, the LDA algorithm can use the traffic incident text data described by natural language to fit several topic models. All of the text data can be matched with these topic models to obtain the probability of each text belonging to a topic. Based on this, we can input this probability as a new feature into the total incident information feature. Then, the LSTM and Bi-LSTM networks are used to construct a deep fusion network for the regression prediction of the traffic incident duration. The construction of the proposed hybrid deep learning model is shown in Figure 3.

It mainly includes eight modules: (1) natural language data and traffic incident variables preprocessing module; (2) LDA topic model module; (3) Bi-LSTM layer; (4) LSTM layer; (5) connect layer; (6) fully connected layer; (7) dropout layer; and the (8) regression layer.

First, in the whole model framework, the traffic incident information is divided into two categories. One is the text data described by natural language, and the other is related to the traffic feature variables. These two types of data are preprocessed separately, and the preprocessing of the text data mainly includes the following points: (1) Convert the text data to lowercase; (2) tokenize the text; (3) erase punctuation; (4) remove a list of stop words such as “and”, “of “, and “the”; (5) remove words with two or fewer characters and words with 15 or greater characters; and (6) lemmatize the words. The main preprocessing method for traffic feature variables is to delete part of the missing data samples and normalize the data samples to eliminate the influence between different dimensions. The normalization formula is as follows.

(8) $$\lambda_{new}=\frac{\lambda_{old}-\lambda_{\min}}{\lambda_{\max}-\lambda_{\min}}$$

where $\lambda_{new}$ is the standardized variable; $\lambda_{old}$ is the original variable; $\lambda_{\max},\lambda_{\min}$ are the maximum and minimum values of the original variables, respectively.

Then, the preprocessed text data are input into the LDA topic model. It is worth noting that the LDA model needs to give the number of fitted topics in advance, and the appropriate number of topics can balance the interpretability and operation efficiency of the topics. The number of topics selected in this study was 14, which is explained in the experimental results.

All features are now complete. The Bi-LSTM layer will accept variables from the text, and traffic incident related variables will be input into the LSTM layer. The principles and formulas of the Bi-LSTM and LSTM were mentioned in the previous subsection. Subsequently, the connect layer combines the information output by the Bi-LSTM and LSTM and inputs it to the full connection layer. This part of the integration is realized by the function connectlayer in MATLAB.

After the full connection layer, we added a dropout layer to reduce overfitting to improve the prediction performance of the model. The dropout technique randomly drops out hidden neurons from the networks. Neurons can be more robust and insensitive by cooperating with a subset of randomly selected neurons. This can improve the generalization ability and prediction performance of deep learning networks on unseen data. Equations (9) and (10) show the calculation of the dropout layer.

(9) r∼Bernoulli(p)

(10) output=r∗input

where r is a random variable donating by Bernoulli distribution, which takes 1 with probability p and 0 with probability 1−p.

Finally, the regression layer computes the half-mean-squared-error loss for regression tasks. The regression layer returns a regression output layer for a neural network as a “RegressionOutputLayer” object.

The prediction accuracy of the neural network depends largely on the setting of the model parameters. However, in terms of previous studies, there was no clear formula or theory to obtain how the specific network parameters were set to the optimal. Some relied on some empirical formulas obtained from previous studies to obtain a relatively reasonable range of parameters, otherwise some parameter optimization techniques were used to improve the model accuracy such as grid search and Bayesian optimization. The prediction method proposed in this study involves multiple parameters including the number of hidden layer units of the LSTM and Bi-LSTM, the number of fully connected layer units, the learning rate of the network, the dropout ratio, the type of the solver, and the number of topics of the LDA model. The specific parameter settings are shown in Section 3.3.

## 3. Experiments

In this section, Section 3.1 discusses the U.S.-accident dataset collected from the Internet. Section 3.2 introduces the evaluation indices of the model. Section 3.3 will clarify the comparison algorithms used in the paper and the parameter settings of all methods.

### 3.1. Data Description

The experimental dataset is publicly available in [11]. The developers of these data applied it to the traffic accident prediction research, where each accident record consists of a variety of intrinsic and contextual attributes such as the location, time, natural language description, weather, period-of-day, and points-of-interest (POI). They collected the streaming traffic data using two real-time data providers, namely “MapQuest Traffic” and “Microsoft Bing Map Traffic”, whose APIs broadcast traffic events (accident, congestion, etc.) captured by a variety of entities—the U.S. and state departments of transportation, law enforcement agencies, traffic cameras, and traffic sensors within the road-networks. From February 2016 to March 2019, a total of 2.25 million traffic accidents were collected covering the United States. This year, the data have been updated to 2020.

**Figure 4:**
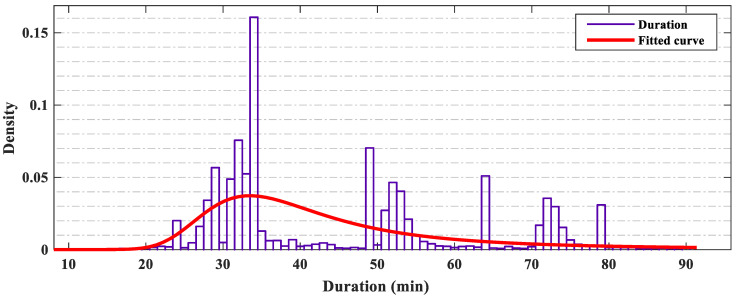
The distribution of the incident duration.

In this paper, we selected the latest part from 1 March 2020 to 30 May 2020, a total of 78,317 traffic incidents. Incident information includes the date and start–end time of the incident, incident ID, severity, affected lane length, location, weather, POI, and incident process described by natural language. At the same time, various traffic incident information is different in terms of the acquisition time. For example, the location of the incident and the weather at that time are often known at the first time. Therefore, we divided these information features into three phases according to the acquisition time. Table 1 details the relevant incident variables used in the study. According to the literature research [9], when the duration of the traffic incident is more than 90 min, the prediction accuracy of various models will be significantly reduced. This part of the samples is more random and unpredictable, and the amount of data is very scarce. Therefore, the scope of this study was within 90 min, and Figure 4 shows the Burr distribution of the data. It can be seen from Figure 4 that the proportion of incidents with a duration of less than 20 min was very small, and the data were concentrated between 20 and 60 min. After 80 min, the samples were quite rare, but the data from about 40 min suddenly decreased. The possible reason is that the data sensor records a part of the data at about 40 min into 35 min, forming a peak at 35 min. The overall fitting curve conformed to the previous expectation of the traffic incident duration.

### 3.2. Evaluation Criteria

To evaluate the performance of the applied models, the root mean square error (RMSE), the mean absolute error (MAE), and the mean absolute percentage error (MAPE) were applied as measures in this study. The formulas of the two criteria were defined as follows:

(11) $$RMSE=\sqrt{\frac{1}{N}\sum_{i=1}^{N}{\left(y_{i}^{o}-y_{i}^{p}\right)}^{2}}$$

(12) $$MAE=\frac{1}{N}\sum_{i=1}^{N}\left|y_{i}^{o}-y_{i}^{p}\right|$$

(13) $$MAPE=\frac{1}{N}\sum_{i=1}^{N}\left|\frac{y_{i}^{o}-y_{i}^{p}}{y_{i}^{o}}\right|$$

where N is the total number of samples in the testing dataset; $y_{i}^{o}$ and $y_{i}^{p}$ are the observed duration and the predicted duration of the ith sample, respectively.

### 3.3. Comparison Methods and Parameters Setting

To evaluate the effectiveness of the proposed model for the prediction of traffic incident duration, several conventional models, namely, the long-short term memory network (LSTM), support vector regression (SVR), single long-short term memory network based on the LAD algorithm (LDA-LSTM), and single bidirectional long-short term memory network based on the LAD algorithm (LDA-BiLSTM) were implemented as benchmark models in this study. In addition, we have also noticed the research results of some scholars in recent years on the prediction of traffic incident duration. In 2021, a deep learning method based on LSTM and MLP was used to dynamically predict the duration of urban expressways [34]. Banishree et al. studied the estimation error bars with the incident duration prediction using the Bayesian optimized support vector regression model [27]. In 2022, an ensemble learning model with multiple clustering was developed. Specifically, the K-means clustering method was used as a bootstrapping technique in the ensemble learning approach, with the individual models based on the artificial neural network model and random forest regression model [36].

Four benchmark models and three advanced algorithms in recent years were used as the comparison algorithms in this study. The specific parameters of these algorithms are shown in Table 2. In order to ensure the performance of the comparison methods, the parameters of these methods were set and optimized according to the corresponding literature. Similarly, in order to assure the fairness of comparison, all methods were set under the same sample database. The specific sample input form was also set according to the form mentioned in the literature. The prediction model proposed involves many parameters. Therefore, based on a large number of experiments, the recommended parameter settings are given for reference only.

## 4. Results and Discussion

In this study, all traffic incident samples were divided into the training set and test set, with a ratio of 7 to 3. The experiment was repeated five times, and the average error of all experiments was calculated as the test result. The number of topics with the LDA topic model is discussed in Section 4.1. Section 4.2 discusses the performance of the proposed method and the comparison method. In Section 4.3, a phased sequential prediction model is proposed with incomplete information. In Section 4.4, we provide a general discussion of the results including a comparison with previous studies.

### 4.1. Number of Topics

The LDA model training takes k (number of latent topics) as a hyperparameter. However, it is challenging to select the optimal number of latent topics to extract good quality and meaningful topics out of the given textual data. To decide on a suitable number of topics, we can compare the goodness-of-fit of the LDA models fit with varying numbers of topics and evaluate the goodness-of-fit of an LDA model by calculating the perplexity of the test documents. The perplexity indicates how well the model describes a set of documents. A lower perplexity suggests a better fit. Choosing an appropriate number of topics can minimize perplexity, but this is not the only consideration: models fit with a larger numbers of topics may take longer to converge. If the optimal number of topics is high, then we might want to choose a lower value to speed up the fitting process.

**Figure 5:**
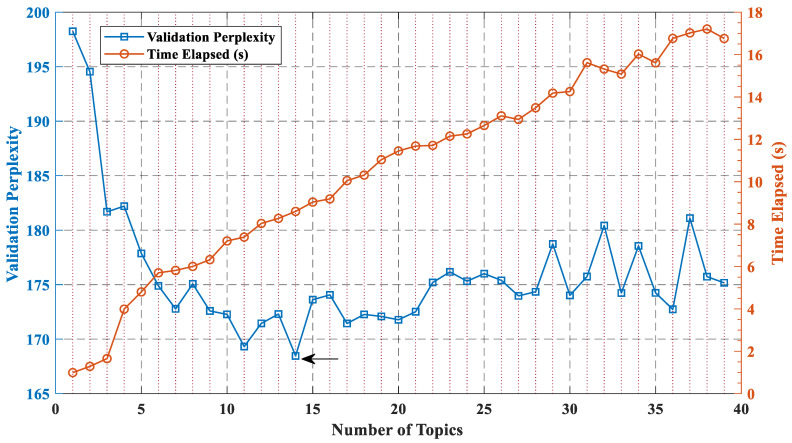
The validation perplexity with various numbers of topics.

Figure 5 shows the relationship between the number of topics and the perplexity of the test documents. It can be seen from the figure that this experiment tested the goodness-of-fit of the LDA model with the number of topics from 1 to 39. The blue line represents the perplexity of the model. The lower the value, the smaller the perplexity. The red line shows the model fitting time, with a maximum of 18 s and a minimum of one second. Therefore, the selection of the number of topics is still based on minimizing the perplexity. As shown by the arrow in the figure, the LDA model had the lowest perplexity when the number of topics was 14 as a parameter for this experiment.

**Figure 6:**
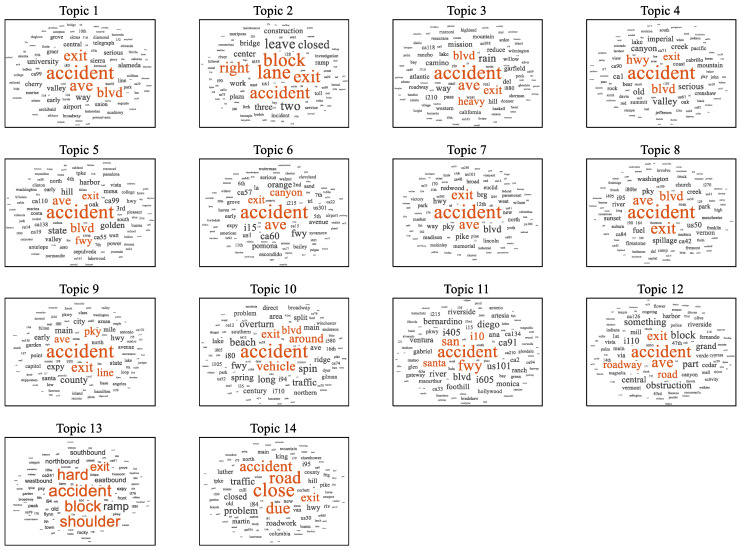
The word clouds of the LDA topics.

The LDA model can input untrained text data into the model and obtain the probability that the text belongs to a certain topic. Figure 6 is a visual word cloud for the generated topic that helps explain the differences and connections between topics. The important words for each topic are highlighted in the word cloud, and the larger the font, the more important the word. It can be seen that accident is highlighted in each topic because this study was regarding traffic incidents, which is an obvious result. In addition, each topic had its own characteristic words. For example, topic 14 can be explained as the lane being closed due to traffic accidents. Topic 2 can be explained as the blockage on the right side of the road caused by an accident at the road exit. Furthermore, it can be found that the meanings of some topics are similar or related, and these topics are not independent. For each traffic incident, it can be given the probability that it belongs to the 14 topics, and 14 characteristic variables are obtained as the input variables of the deep learning network.

### 4.2. Comparison with Other Models

Table 3 shows the RMSE, MAE, and MAPE of the seven comparison methods above-mentioned. First, the error of the proposed method was the smallest on the RMSE and MAE, and the error of the conventional LSTM and SVR methods was larger. After using the LDA algorithm, the error of the single LSTM and single Bi-LSTM network was reduced compared with the ordinary LSTM, but still higher than the proposed hybrid network. This result shows that the natural language features extracted by the LDA algorithm has a certain positive effect on the prediction of the traffic incident duration, and the hybrid network with feature fusion is superior to the single network structure. It can be seen that it is feasible and reasonable to propose a hybrid network to predict the duration.

Moreover, compared with the three advanced methods, the prediction accuracy of our proposed method was the best, except that the MAPE of the MLP-LSTM and the proposed algorithm were both 0.31. The RMSE of the EMC method was the largest, which may be due to the poor performance of the ANN, the individual learner in ensemble learning, but the gap with the proposed method was 1.39%, which was better than that of SVR. MLP-LSTM had the second highest performance after our proposed prediction model. The performance of SVR was unexpected. Although the RMSE was high, the MAPE was the best among all of the algorithms. Early machine learning algorithms still have the potential to grow.

**Figure 7:**
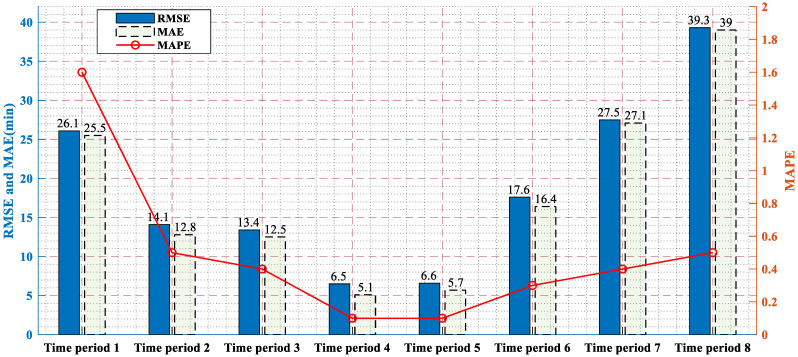
The error of the proposed prediction model in each time period.

In order to further study how the proposed prediction model works on traffic incidents, we divided the duration into eight periods, namely (0, 20], (21, 30], (31,40], (41, 50], (51, 60], (61, 70], (71, 80], (81, 90]. Figure 7 shows the error of the prediction model in each period. The blue bar and green bar represent the RMSE and MAE, respectively. The red broken line is MAPE, whose axis is on the right. It can be seen from the figure that time period 2 to time period 6, namely 21 min to 70 min, was the preference group of the prediction model. When the duration was about 10 min, the error was higher than the overall average error of the model, but this part of the sample only accounted for 0.45%, and the sample size was too small to support the model training accuracy. The same problem also occurred in time period 8, namely 81–90 min, and its sample proportion was 1.12%. However, the proportion of samples with a duration of 71–80 min was 14.5%, and its error was 10% higher than the overall average, which means that the prediction model had heterogeneity at the beginning of the traffic incident with a duration greater than 70 min. In other words, some information on the traffic incidents can not be collected. When the duration was more than 70 min, this part of information started to influence the duration more significantly than the collected information.

In general, the proposed model had the overall average prediction accuracy for 83.93% of the samples from time period 2 to time period 6, which allows for the traffic decision-makers to judge the impact of traffic incidents on the whole and improve the congestion after the incident.

### 4.3. A Phased Sequential Prediction Model

So far, all studies in this paper were based on the condition that all traffic information is available, which is not realistic. There are many reasons such as equipment failure, which will cause information loss; relevant parameters are updated as the time progresses; and the information acquisition time. This phenomenon is called the incomplete information condition.

However, it is too late for traffic decision-makers to act when all of the information is available. Therefore, considering the actual situation, a sustainable duration prediction model is essential. Then, based on the above reasons for incomplete information, this study regarded information acquisition as a dynamic updating process. Because the characteristic variables collected by the database used in this study cannot be sorted according to the time of acquisition of each variable, we divided the variables into three phases. Variables at the same phase were considered to be obtained simultaneously, which can be seen in Table 1. When the incident state is in one-phase, the information for the two- and three-phases is unavailable. When the incident state develops to the two-phase, the three-phase information is unknown.

Based on the above assumption, we introduced the phased sequential prediction model.

Step 1: The training set is clustered by K-means to obtain the centroid coordinates of each cluster.

Step 2: The training set is put into the proposed hybrid deep learning network to train, and then the trained prediction model is saved.

Step 3: When the state develops to the one-phase, the one-phase test sample will be typed to a class label based on a partitioned cluster.

Step 4: The missing two-phase and three-phase test data for the one-phase are replaced by the centroid coordinates of the cluster.

Step 5: The updated test samples are predicted by the trained model.

Step 6: When the state develops to the two-phase, operations similar to Step 3 and Step 4 are repeated to replace the missing data, and then the updated test samples are predicted.

Step 7: When the state develops to the three-phase, all information is available and can be predicted directly.

It is worth mentioning that in this study, the variables are divided into phases, and each phase contains a different number of variables. If other datasets can define the acquisition time of each feature, then the model is developed into a dynamic continuous model.

**Figure 8:**
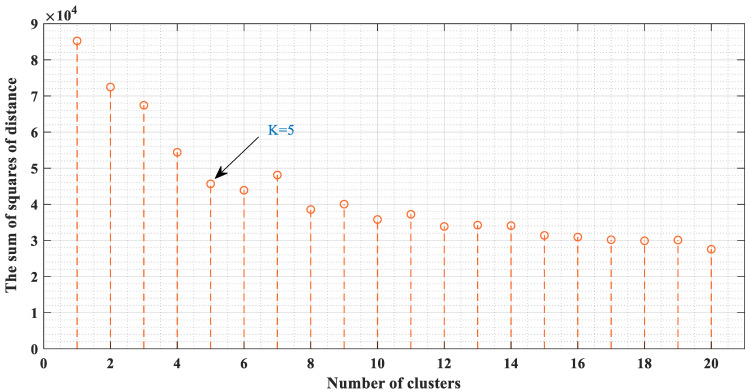
The sum of the squared distances with the number of clusters.

This phased sequential prediction model has one parameter that needs to be given in advance by the training set, that is, the number of K-means clusters. Figure 8 shows the sum of the squared distances from each point of the cluster to the centroid with different cluster numbers. As shown by the arrow in Figure 8, when the number of clusters is 5, the decreasing trend slows down and tends to be stable. Therefore, we choose 5 as the parameter value of K-means.

Table 4 shows the performance of the prediction model in different phases, using RMSE, MAE, and MAPE to evaluate. As we expected, the prediction error should be reduced as the time progresses and more relevant details are added to the dataset. At the same time, compared with the ordinary LSTM using all data in Table 3, a one-phase prediction model using partial data is superior. This proves that the phased sequential prediction model is feasible, reasonable, and reliable.

### 4.4. Discussion

In the previous subsections, we showed the experimental results of several important points of this study, and provide some analysis and discussion on the results. In this subsection, we will complement some discussion and talk about the current limitations of this study.

First, from the aspect of natural language processing, we chose the LDA topic model, which is a very mature language processing technology and has been applied in many fields. In other words, some more novel algorithms have been proposed in the field of language processing. Some scholars have also applied them to the traffic incident duration prediction. In [9], the BERT algorithm was used for natural language processing, and then the prediction performance of several conventional machine learning algorithms was compared. The MAE of the best model reached 11.16 min, which was close to the performance of our proposed algorithm, although the data used by the two were different. This was not only the limitation of this study, but also the potential for development. Replacing this with more excellent language processing technology is expected to improve the overall performance.

Second, in the three recent studies compared with the proposed algorithm in Table 3, one was an ensemble learning algorithm based on clustering, another was a support vector machine regression based on Bayesian optimization, and the third one was a multi-layer hybrid model of LSTM and MLP. Compared with these three types of algorithms, it was found that the LSTM and MLP multi-layer hybrid model with similar architecture to our proposed algorithm had better performance than the other two types of algorithms, and was only slightly lower than our proposed algorithm. From the rapid development of deep learning in recent years, the neural network structure may be more advantageous on large-scale datasets. This result is in line with our expectations. In previous studies [24], the MAE of the algorithm was 14–36 min, and the MAPE was 22–33%, which was consistent with our experimental results.

Finally, we proposed a phased sequential prediction model in Section 4.3. The premise is that the acquisition of information is regarded as a dynamic updating process, and the characteristic variables are divided into three phases. The variables at the same phase were obtained simultaneously. The results show that the model error is lower as the information is updated. Although the experimental results were as expected, it is more desirable to establish a continuous dynamic prediction model. In the future, if we can define the order of acquisition of this variable, we can expect to achieve this goal.

## 5. Conclusions

The reliable prediction of the traffic incident duration is of great significance for traffic decision-makers to formulate timely and effective measures. For various practical reasons, traffic incident information is not always available, and this incomplete information will be updated with the development of the incident state. Therefore, this paper studied some traffic incidents that occurred across the whole of the United States in 2020.

Based on the text data described by natural language and sensor data, a hybrid deep learning model was proposed to predict the incident duration. In this model, the LDA topic modeling module extracted natural language as feature variables for network learning, and the Bi-LSTM and LSTM hybrid deep learning network was used for prediction. The RMSE, MAE, and MAPE were used to evaluate the model performance. The results showed that the model had the best prediction effect when the duration was 20 min to 70 min. Then, seven different methods including four benchmark models and three advanced algorithms in recent years were compared, and the proposed model’s RMSE and MAE were the lowest. Finally, this study proposed a phased sequential prediction model, which could predict the duration of the incident according to the development phase of the incident. The experimental results were credible and reasonable.

In the future, other datasets will be considered to develop the phased sequential prediction model into a dynamic continuous prediction model. At the same time, traffic incidents have spatio-temporal correlation characteristics, and how to use this feature in the dynamic model will also be the research focus in future studies.

## Figures and Tables

**Table 1:** The feature variables used in this study.

Categories	No.	Variables	Type	Coding
One-phase	1	Temperature (F)	Continuous	Numeric
	2	Wind Chill (F)	Continuous	Numeric
	3	Humidity (%)	Continuous	Numeric
	4	Pressure (in)	Continuous	Numeric
	5	Visibility (mi)	Continuous	Numeric
	6	Wind Speed (mph)	Continuous	Numeric
	7	Weather Condition	Binary	Normal = 0, Severe = 1
	8	Amenity	Binary	Not exist = 0, Nearby exist = 1
	9	Bump	Binary	Not exist = 0, Nearby exist = 1
	10	Crossing	Binary	Not exist = 0, Nearby exist = 1
	11	Give Way	Binary	Not exist = 0, Nearby exist = 1
	12	Junction	Binary	Not exist = 0, Nearby exist = 1
	13	No Exit	Binary	Not exist = 0, Nearby exist = 1
	14	Railway	Binary	Not exist = 0, Nearby exist = 1
	15	Roundabout	Binary	Not exist = 0, Nearby exist = 1
	16	Station	Binary	Not exist = 0, Nearby exist = 1
	17	Stop	Binary	Not exist = 0, Nearby exist = 1
	18	Traffic Calming	Binary	Not exist = 0, Nearby exist = 1
	19	Traffic Signal	Binary	Not exist = 0, Nearby exist = 1
	20	Sunrise Sunset	Binary	Night = 0, Daylight = 1
	21	Civil Twilight	Binary	Night = 0, Daylight = 1
	22	Nautical Twilight	Binary	Night = 0, Daylight = 1
	23	Astronomical Twilight	Binary	Night = 0, Daylight = 1
Two-phase	24	Description of incidents	Natural language	Document
Three-phase	25	The severity of the incidents	Categorical	Four levels = (1, 2, 3, 4)
	26	Influential distance (mi)	Continuous	Continuous
Response	27	Duration	Continuous	Continuous

**Table 2:** The hyperparameters of all methods.

Methods	Hyperparameters
The proposed method	Initial learning rate (0.001); Adam optimizer; Number of neurons in hidden layers (LSTM = 128, Bi-LSTM = 64, Fully connected = 128); Dropout rate (0.4); Number of LDA topics (14).
LSTM	Number of neurons in hidden layers (LSTM = 128).
SVR	The linear kernel function.
LDA-LSTM	The same as our method.
LDA-BiLSTM	The same as our method.
MLP-LSTM	Number of neurons in hidden layers (LSTM = 128); MLP contains two hidden layers.
BSVR	Parameter optimized by Bayesian.
Ensemble Model Based on Clustering (EMC)	The K-means clustering (K = 3, 4, 5); Individual models based on the artificial neural network model.

**Table 3:** The performance of different methods for the incident duration prediction.

Methods	RMSE	MAE	MAPE
The proposed method	15.72	13.12	0.31
LSTM	16.99	14.72	0.35
SVR	17.33	13.45	0.28
LDA-LSTM	16.25	13.98	0.34
LDA-BiLSTM	16.23	13.87	0.33
MLP-LSTM	16.21	13.56	0.31
BSVR	16.78	14.69	0.35
EMC	17.11	14.24	0.32

**Table 4:** The performance of the phased sequential prediction model in different phases.

Incident States	RMSE	MAE	MAPE
One-phase	16.59	14.37	0.34
Two-phase	16.33	14.03	0.33
Three-phase	15.72	13.12	0.31

## Data Availability

The datasets used and/or analyzed during the current study are available from the corresponding author on reasonable request.
